# A human-like EEG signature of cognitive control in the domestic dog

**DOI:** 10.1007/s10071-026-02096-8

**Published:** 2026-08-11

**Authors:** Ivaylo Borislavov Iotchev, Anna Kis, Márta Gácsi

**Affiliations:** 1 Momentum” Psychobiology Research group, HUN-REN Research Centre for Natural Sciences, Institute of Cognitive Neuroscience and Psychology, Budapest, 1117 Hungary; 2 Department of Ethology, ELTE Eötvös Loránd University, Pázmány Péter sétány 1/c, Budapest, 1117 Hungary; 3 HUN-REN–ELTE Comparative Ethology Research Group, Budapest, Hungary; 4 NAP Canine Brain Research Group, ELTE Eötvös Loránd University, Budapest, Hungary

## Abstract

In humans, theta (θ) band activity (defined as 4–8 or 5–7 Hz), measured over the frontal midline of the scalp, is an important EEG correlate of voluntary and conscious self-control. Theta waves recorded over the frontal lobes can therefore implicate neocortical control mechanisms. Other likely physiological markers include alpha and beta power, and more recently, a flattened 1/f slope. In the domestic dog, inhibitory self-control is extensively studied, but mostly through behavioural tests. Here, we present a first inquiry into possible EEG correlates of cognitive control in family dogs by comparing short (~ 30-second-long) EEG recordings from two conditions: passive wakefulness (baseline) versus a delayed gratification challenge (test). These conditions were recorded in a random sequence within the same session, under overall identical settings. In total, we collected 226 short recordings from fourteen dogs. We observed an increase in neural activity (test > baseline) that resembles human cognitive theta in frequency range (4–8 Hz) and which is similarly more pronounced over the frontal midline. Another important finding is a reduction in broadband power and the flattening of the spectrum, which implicates the work of fronto-parietal connections. While these markers do not differentiate between different facets of cognitive control, our capacity to measure them in awake dogs sets the stage for further EEG-based inquiries into canine cognition.

**Supplementary Information:**

The online version contains supplementary material available at https://doi.org/10.1007/s10071-026-02096-8.

## Introduction

‘Cognitive control’ refers to those mechanisms of the mind-brain (Eisma et al. 2021), by which behaviour is made to align with one’s goals and plans. Cognitive control allows information to be prioritized, structured, or ignored. It underlies the willed and conscious selection (van Schie et al. 2022) and suppression (Yamanaka and Yamamoto 2010) of action choices. Cognitive control is impacted in neuropsychological conditions such as ADHD, leading to impairments in everyday life (de Zeeuw et al. 2012; Shaw et al. 2006). Such relationships have also been described in the dog model of ADHD (Kovács et al. 2025).

In humans, cognitive control functions are localized in the (pre)frontal lobes (Ball et al. 1999; Swick et al. 2008; Zavala et al. 2018). The anatomy of the dog brain includes analogues of these structures (Cook et al. 2016b; Czeibert et al. 2019; Szabó et al. 2023), but when and how the animals’ behaviour is under prefrontal control is not thoroughly researched. Most of the findings in dogs are derived from behaviour - reviewed in Olsen (2018), overwhelmingly from studies on inhibitory control (Bray et al. 2014; Brucks et al. 2017; Cook et al. 2016b; Olsen 2022), i.e. the ability to suppress impulses, and task-irrelevant, or wrong responses. To date, only one study has linked this capacity to a hemodynamic response in the dog’s frontal lobes (Cook et al. 2016b), but there are at least three reasons to expand our search for the neural substrates of canine cognitive control.

First, the many-to-one mapping problem in comparative cognition (Taylor et al. 2022) highlights a general problem with observational data: most behaviours can be achieved through different mechanisms or strategies. Response inhibition is no exception to this rule. It has been shown, for instance, that the cerebellum can inhibit motor responses in the absence of an intact neocortex (Hesslow 1994). Another subcortical structure hypothesized to play a crucial role, specifically in the regulation of dogs’ behaviours, is the subthalamic nucleus (Medenica et al. 2025). In humans, the contributions of the frontal lobes and subthalamic nucleus are not mutually exclusive (Zavala et al. 2018), however, it is common to observe structures that operate both within a hierarchy and independently. Since only neocortical inhibition is linked to voluntary and cognitive control of motion (van Noordt et al. 2022; Yamanaka and Yamamoto 2010), a closer examination of the neural correlates of response inhibition, especially when they implicate frontal lobe involvement, can highlight the level of cognitive engagement during action suppression.

Second, the behavioural data to date suggest that within a dog, the successful use of inhibitory control varies across tasks and contexts (Bray et al. 2014; Brucks et al. 2017). To understand which situations prompt prefrontal control (as opposed to subcortical strategies), it is important to pair behavioural and neural measures in canine cognitive experiments. To date, the only study to do so (Cook et al. 2016b) used a variation of the human Go/No-Go paradigm, with dogs lying motionless in an MRI scanner. However, situations closer to everyday challenges, and thus with appropriate ecological validity, have not been examined in dogs using neuroimaging.

Third, while fMRI can most directly and reliably demonstrate the localization of task-relevant brain activity, follow-up research is hindered by the strong sensitivity of this technique to motion artefacts and its high cost of implementation (Montgomery 2023). While restricted motion is the ideal condition for all neuroimaging methods, EEG is somewhat more forgiving and has well-established cleaning protocols, e.g. for eye-movement artefacts (Ronca et al. 2024). EEG is also implemented at a lower cost (Montgomery 2023) and does not require the animals to undergo lengthy training before they can be measured (Kis et al. 2014). All of these facts make it more likely to establish an extended series of cognitive control studies in dogs, if EEG concomitants of prefrontal control are identified.

In humans, elevated power in the theta band, defined either as 4–8 Hz (Eisma et al. 2021; van Noordt et al. 2022), or more narrowly, 5–7 Hz (Inanaga 1998; Jadeja 2021), is linked to various types of cognitive control (Cavanagh and Frank 2014; Eisma et al. 2021; Inanaga 1998). This shared expression in the surface EEG signal, obscures the fact that different functions associated with cognitive control belong to different sub-structures within the frontal lobes (Manza et al. 2016; Roelofs et al. 2006; Swick et al. 2008; Vendrell et al. 1995). A deeper set source of theta waves is the hippocampus (Klimesch 1999; Nuñez and Buño 2021) whose synchronization with frontal structures underlies some facets of cognitive control (Sigurdsson and Duvarci 2016). Due to volume conduction (Buzsáki et al. 2012) cognitive theta is mostly pronounced over the same frontal recording sites, regardless of the precise sub-structure involved (Eisma et al. 2021; Inanaga 1998). This contrasts with a more diffuse and widespread increase in theta power, which can be observed during drowsiness and sleep pressure (Britton et al. 2016; Snipes et al. 2022). There is a well-established link between theta power and response inhibition in particular (Manza et al. 2016; Swick et al. 2008; van Noordt et al. 2022; Yamanaka and Yamamoto 2010; Zavala et al. 2018). It is thereby crucial that only prefrontal theta is linked to response inhibition, whereas theta recorded from the subthalamic nucleus is instead associated with response initiation (Zavala et al. 2018). This suggests that theta observed during action suppression will indicate neocortical control of behaviour more reliably than theta observed during (re)active states.

Since theta, linked to higher cognitive functions, is consistently found at the frontal midline of scalp EEG recordings in humans (Eisma et al. 2021; Inanaga 1998; van Schie et al. 2022), regional differences in theta power changes may prove crucial in analysing canine EEG for analogues of cognitive theta. There are currently only two active midline electrodes in most dog EEG experiments (Kulgod et al. 2025), but they appear sufficient to replicate human-like differences in topography. For instance, a higher number of high-frequency spindles can be counted during slow-wave sleep over the more posterior midline electrode (Iotchev et al. 2019), as also seen in humans (Jobert et al. 1992) and rats (Terrier and Gottesmann 1978).

A few other changes in the EEG spectrum have also been associated with inhibitory control and/or mental effort in humans. These include the beta-frequency band (Hwang et al. 2014; Lu et al. 2024; Zavala et al. 2018), the alpha frequency band (Hummel et al. 2002; Hwang et al. 2014; Lu et al. 2024), and even changes in the broadband, i.e. overall spectral power independent of specific bands (Jacob et al. 2021; Lu et al. 2024; Pei et al. 2023). The latter appears to index aperiodic aspects of EEG activity, which, unlike band-specific markers, display a negative relationship with mental effort (Lu 2025). In visualizations of the frequency domain, the aperiodic part of the signal appears as a slope from which frequency-specific signals stand out as separate, localized peaks (Gerster et al. 2022). This part of the signal is therefore sometimes referred to as ‘aperiodic slope’ or ‘1/f slope’. While some authors calculate the intercept and steepness of the slope, Lu (2025) concludes that aperiodic broadband power (≤ 30 Hz) is by itself the single most successful correlate of mental effort across studies.

Most of these EEG signatures, except theta power, do not specifically reflect the role of the (pre)frontal lobes. Alpha power is usually interpreted as indexing inhibitory mechanisms which are being ‘recruited’ locally, following a central decision to inhibit a response (Hummel et al. 2002; Hwang et al. 2014), while a drop in overall power across bands likely reflects activity in a more distributed, fronto-parietal network (Lu 2025).

In the present work, we intend to monitor dogs’ EEG signals across all known physiological markers of cognitive control, with a strong emphasis on frontal midline theta as an indicator of frontal lobe involvement. We devised a delayed gratification challenge for dogs, which required response inhibition towards the target, since it was visible and smellable, and remained within reach during the delay period. In addition, this challenge may invoke other aspects of cognitive control, including attention (Sauseng et al. 2007; Ullsperger et al. 2014), response preparation and anticipatory control (Chiew and Braver 2014), and response conflict (Carter and Botvinick 1999; Nigbur et al. 2011; Ullsperger et al. 2014). However, the areas linked to different types of cognitive control are localized close to each other in the frontal lobes (Carter and Botvinick 1999; Roelofs et al. 2006; Swick et al. 2008; Vendrell et al. 1995) and are together expressed as a more anterior theta-power elevation (Eisma et al. 2021; Inanaga 1998). Therefore, a primary aim of this study was to establish if a situation with an objectively stronger need for self-control would coincide with markers of frontal lobe involvement in the EEG, while subcortical control mechanisms or simply acknowledging the presence of reward are not expected to affect scalp measured theta or aperiodic broadband activity (Hesslow 1994; Marco-Pallares et al. 2015).

To this end, we compared the EEG from this test condition with representative baseline recordings from within the same session, and therefore under the same overall conditions (same room, same time of day, same humans present, i.e. owner and experimenter). Since the test condition was linked to a need for self-inhibition, we expected to see higher frontal midline theta, and possibly, but less localized elevations in the alpha and beta bands during test recordings compared to the baseline. Because recent evidence reviewed by Lu (2025) suggests that mental effort leads to an overall decrease in broadband power, we further hypothesized that band-specific correlates of cognitive control would be more expressed in measures of relative or slope-corrected power, while broadband effects were studied using absolute power cleaned from oscillatory components (Gerster et al. 2022).

## Methods

### Ethics

By Hungarian law, non-invasive and harm-free studies with animals do not count as animal experiments. Ethical permission for our non-invasive project was issued by the Hungarian Scientific and Ethical Committee of Animal Experiments (PE/EA/2019-5/2017). All owners provided written consent to participate with their dogs in the study. They were informed of the type of data we collect, how the data is to be kept safe and anonymous, and were permitted to withdraw from the study at any point.

### Subjects

Fourteen dogs were measured in this study with 10 to 21 trials per subject (totalling *N* = 226 measurement points). The age, sex and breed of the subjects, as well as participation in previous experiments involving non-invasive EEG, are reported in Table 1. Dogs were selected on the basis of having received training in accordance with the demands of the task, i.e. they were trained to abstain from eating a treat or grabbing a toy if the owner forbid it. We did not restrict inclusion in the study based on age, breed or prior experience with EEG, but we documented the individual differences for future reference and use (e.g. meta-analyses). Sample size was modelled after small-scale example studies to ensure alignment with the three *Rs* - Replacement, Reduction, Refinement (Russell and Burch 1959). The number of dogs recruited falls within the range of studies that served as a point of reference, either because they investigated theta power effects in humans on the level of single trial data (Yamanaka and Yamamoto 2010) or constitute examples of measuring EEG^1^ in dogs (Iotchev et al. 2020). In these example studies, each counting two to three separate experiments, the sample size ranged from 9 to 19 participants (*N* = 9 to 15 in Yamanaka and Yamamoto, *N* = 13 to 19 in Iotchev et al.).

**Table 1:** Identification codes and demographic data of the participating dogs

Dog ID	age (years)	sex	breed	prior EEG experience
BSS	6	male	Kelpie	yes
FMG	3	male	Cairn Terrier	yes
DMG	1	male	Tervuren	yes
JZB	6	male	mixed breed	yes
VLZ	1	female	mixed breed	yes
ASS	1	female	mixed breed	yes
KAJ	2	female	Havanese dog	no
GAG	8	female	Nova Scotia Retriever	yes
FAG	2	male	Nova Scotia Retriever	yes
CKT	5	male	mixed breed	no
NKT	1	female	Central Asian Shepherd	no
BCC	8	male	mixed breed	no
KJA	6	female	Yorkshire Terrier	no
BAA	6	male	mixed breed	no

### Behavioural paradigm

All dogs were allowed to explore the room and meet the experimenter prior to the experiment. We did not tightly control how the owners managed their dog, but any peculiarities in the conduct of the dog or owner during the session were captured in a notebook and for twelve out of fourteen dogs, the sessions were also video-recorded.

For each dog, all recordings took place within the same experimental session to ensure that similar factors characterize both experimental conditions: same room, same number of people present (owner and one experimenter), same time of day.

Two behavioural conditions were the source of our EEG recordings; they will be referred to below as ‘baseline’ and ‘test’. For the safety of our recording equipment and to minimize movement artefacts, it was necessary to somewhat restrict the dogs’ movement in both conditions. Therefore, prior to the experiment, each dog was designated a spot on which to calmly reside. This might have required some self-control from the dogs (under the guidance of the owners), but it was a constant across both conditions. Only in the test condition was an additional delayed gratification challenge added; the dogs were awaiting permission to approach and take a treat placed in front of them. Treat identity was individually adjusted to make sure that every dog was challenged with a treat of their liking, since in humans, inhibitory control in the absence of emotionally salient stimuli requires less theta band elevation (Andreu et al. 2019). The owners chose (or approved) which rewards their dog valued, based on their experience. Two dogs were tested with a toy. For GAG, a toy was used instead of an edible treat. For ASS, only two toy trials were recorded, since the impatience of this dog ended the session after this (ASS increasingly tried to stand up and walk away, and after losing one of the electrodes, we ended the session for the safety of the equipment). The types of delayed reward used for each dog are summarized in Supplementary Table S1.

EEG recordings in the test condition started when the forbidden treat was placed in front of the dog, within its reach, followed by a command, always issued by the owner, instructing the dog to wait. The exact start of the recording coincided with placing the reward in its final position, just before the command was issued. After approximately 30 s of EEG recording, the dog was allowed to eat the treat (or grab the toy) to stay motivated for further trials. EEG recording during test trials was halted before the dog was allowed to approach the reward. Baseline recordings were randomly taken before, between, and after test trials. Random sequences of baseline and test trials were created using simple randomization with equal allocation probability. To match the duration of the recordings from each condition, we aimed for 30 s in both, with minor variation in the final data (standard deviation of 3 s). The exact number of trials per condition and the randomized sequence in which they were recorded is displayed for each dog in Table 2.

**Table 2:** Sequence, number, and type of trials (T = test, B = baseline) for each dog

Dog ID ↓ / Trial number →	1	2	3	4	5	6	7	8	9	10	11	12	13	14	15	16	17	18	19	20	21
BSS	B	T	B	T	B	B	T	B	T	B	T	T	B								
FMG	T	B	T	T	B	B	T	B	T	T	B	T									
DMG	B	T	B	T	T	B	T	B	T	T											
JZB	B	B	B	T	T	B	B	T	B	T	B	T	T	T	B	B					
VLZ	B	B	T	B	T	T	B	B	B	T	T	B	B	T	B	T	B	T	B		
ASS	T	T	B	B	B	T	T	B	B	T	T	T	B	B	B	B	T	T	B	T	B
KAJ	T	T	B	B	B	T	B	B	B	T	T	B	B	T	B	B	T				
GAG	T	T	B	B	B	T	T	B	B	T	T	B	B	T	T	B	B				
FAG	B	B	B	B	T	B	T	B	T	T	B	B	T	T	T	B	B				
CKT	B	B	T	T	B	B	T	T	B	B	T	T	T	B	B	B	T	T			
NKT	B	B	T	T	T	B	B	B	B	T	T	T	T	B	B	T	T				
BCC	B	B	B	B	T	T	B	B	T	T	T	B	B	T	T						
KJA	B	B	B	B	B	T	T	B	B	T	T	B	B	T	T	T					
BAA	B	B	B	T	T	B	B	T	T	B	B	B	T	T	B	B	T	T			

### EEG recordings

**Fig. 1:**
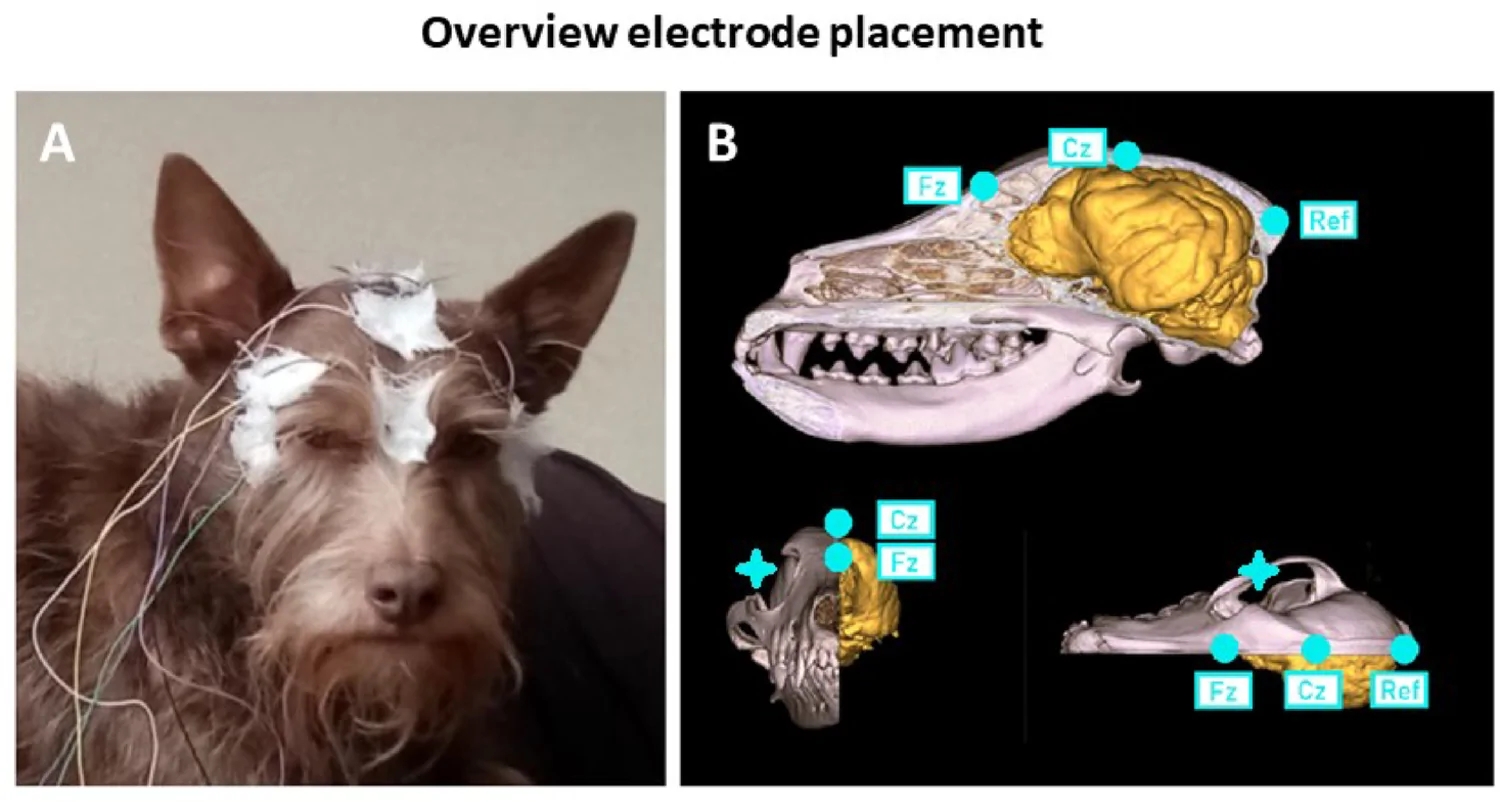
Placement of electrodes, real-life example (**A**) and a 3D model of electrode position relative to the brain of a typical dog, highlighting, in particular, electrodes Fz, Cz, and reference (**B**). The placement of eye-movement tracking electrodes (F7/F8) is indicated unilaterally with a light blue star in the frontal and dorsal view. 3D model by Kálmán Czeibert, used with permission

The set-up used here to record EEG in awake dogs (Fig. 1) is a variation on the one developed for sleep research in this species by Kis et al. (2014). Six gold-coated Ag/AgCl electrodes were attached to the scalp of the dog with EC2 gel (Grass Technologies, USA), keeping the impedance below 15 kΩ. A 30-channel Flat Style SLEEP La Mont Headbox and an HBX32-SLP 32-channel preamplifier (La Mont Medical Inc., USA) were used to collect and pre-process the signals. The occipital protuberance was used to place a reference electrode (Kulgod et al. 2025), and the musculus temporalis for the ground electrode. Signals of interest were collected from the skull midline, with a frontally placed electrode (Fz), between the eyes, and a central electrode (Cz) between Fz and reference. To monitor eye movements, additional two electrodes were placed on the zygomatic arch (F7 and F8).

### EEG post-processing

Prior to filtering the signal, the EEG traces were cleaned for eye-movement artefacts. We chose regression-based cleaning (Ronca et al. 2024) instead of artefact-rejection, since traditional thresholds for artefacts in our experience lead to losing most, if not all, of the signal in dogs. Some authors have even argued completely against cleaning (Delorme 2023), but applying no corrections at all in dog EEG, which is noisier than in humans, is likewise unfeasible. We used the eye-movement tracking electrodes (F7 and F8) as predictors for either Fz or Cz activity, subtracting the explained variance from the raw EEG signal of each midline electrode. To minimize the loss of real signal that may be shared between the midline and eye-movement tracking electrodes, the latter were first low-pass filtered at a passband of 15 Hz (stopband 17 Hz), since the main spectral content of eye-movement artefacts was found to usually not exceed 15 Hz (Ronca et al. 2024).

All filtering of the EEG prior to analysis was done using second-order section Butterworth filters, similar to those in our previous work on dogs’ sleep EEG (Iotchev et al. 2019, 2023). The low-pass boundary was set to 30 Hz (passband) and 35 Hz (stopband). The high-pass boundaries were set to 0.1 Hz (passband) and 0.05 Hz (stopband). The 0.1 to 30 Hz passband range was used here to align with several previous works in humans (Batterink and Paller 2017; Batterink and Zhang 2022; Lyamin et al. 2008; Waterman et al. 1993) and our own previous analyses in dogs (Iotchev et al. 2023, 2024). Crucially, this filter regime was applied only prior to our own method for quantifying spectral power (see detailed below). For spectra obtained with IRASA (Irregularly Resampled Auto-Spectral Analysis) see the default settings of this algorithm (Wen and Liu 2016).

### Frequency-power extraction

Our pilot explorations (shared in the Supplementary) revealed that absolute power was positively ‘correlated’^2^ across all commonly studied frequency-bands (alpha, beta, delta, theta) and that the entire spectrum (≤ 30 Hz) was affected by condition. This is in line with strong changes in the aperiodic slope (Brake et al. 2024) and renders absolute power invalid for testing frequency-specific hypotheses. The remaining options: relative and slope-corrected power present trade-offs. Specifically, slope-correction is becoming more common (Lu 2025), but isn’t free of controversy (Brake et al. 2024; Gerster et al. 2022) and there is different recommendations for how and when to detrend the signals (Brake et al. 2024). Below we therefore detail how relative and slope-corrected power were obtained, and when each was used. Since estimates of frequency-specific power are more sensitive to incorrect modelling of the slope than estimates of aperiodic broadband power (Brake et al. 2024; Wen and Liu 2016), the complementary approaches discussed below only concern the former.

**Fig. 2:**
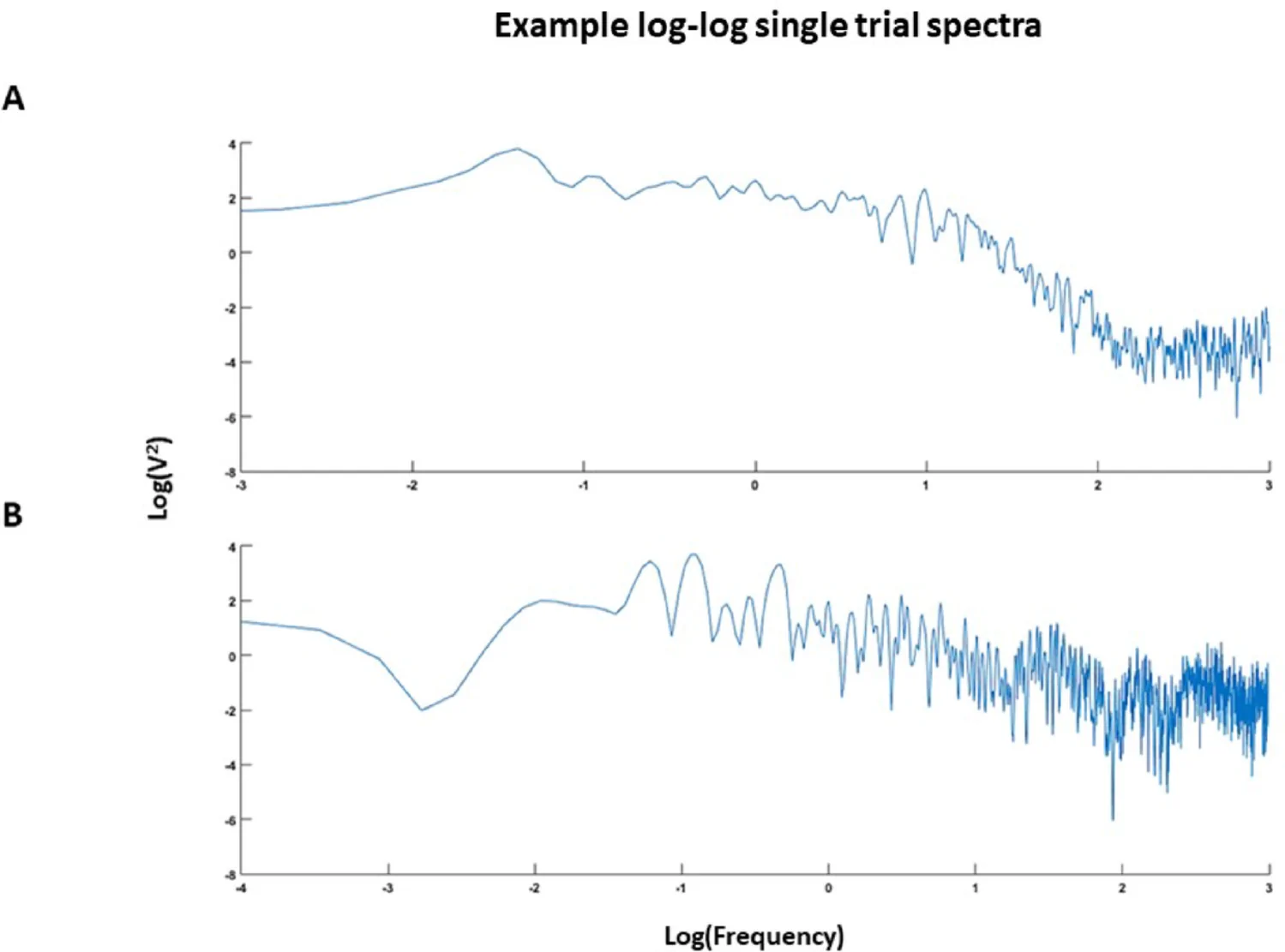
Example log-log view of two frequency-power spectra from single-trials, a baseline recorded in JZB (**A**) and a test trial from KJA (**B**). X-axis: log-transformed frequency, y-axis: log-transformed power

The validity of using 1/f-corrected oscillatory power was assessed visually in line with the recommendations by Gerster et al. (2022) by checking whether the log-log representations of the spectra follow a straight line. This assumption was violated for single-trial data (see examples in Fig. 2). Therefore, accounting for the 1/f slope in our analyses was only applied to data pooled across trials, whereas single-trial data was derived with a variation of our traditional approach to quantifying relative spectral power in dogs (Iotchev et al. 2023; Kis et al. 2017). Our method uses 4-second-long, sliding time-windows with 50% overlap, applied with the *spectrogram.m* function in Matlab. We deviated in only two choices from a previous version of this calculation (Iotchev et al. 2023). First, we increased the frequency resolution to 0.01 Hz (from previously 0.1 Hz). Second, across time-windows and frequency bins, power was median-averaged. The use of medians makes the estimates more robust to artefacts (Bassett Jr 1991) that may have been missed by the automatic cleaning. Each band was defined as previously in the dog (Iotchev et al. 2023; Kis et al. 2017): alpha 8–12 Hz, beta 12–30 Hz, delta 1–4 Hz, and theta 4–8 Hz. To obtain relative power within bands, the median for each band was re-calculated as a fraction of the sum of all four bands.

The IRASA algorithm (Wen and Liu 2016), specifically the *amri_sig_fractal.m* function, was used to obtain raw and separated (into aperiodic and oscillatory) power spectral densities for data which was median-averaged across trials. In order to pool data across trials, it was important to ensure that all spectrograms had the same length and scaling. To achieve this with IRASA, we had to restrict the maximum trial length in the time domain to exactly 30 s. The IRASA outputs for the raw and aperiodic part of the spectrum were first median-averaged across trials for each dog and condition, then subtracted (raw - aperiodic). The resulting residuals were transformed to standard scores, and a threshold of 1 standard deviation was applied to obtain frequency-specific estimates of power which extend beyond 1/f noise. The method for applying a threshold and the cut-off (1 standard deviation) are adopted from a tutorial by Cole et al. (2019). For each band and condition, power above the threshold was integrated (*trapz.m* function in Matlab). Subtraction plus thresholding allowed us to test if and which frequencies depart significantly from the aperiodic slope. This is recommended to ensure that condition differences in frequency power are not artefacts caused by condition-dependent changes in slope steepness (Brake et al. 2024).

Threshold tests on the pooled data followed as a control after condition effect tests on the single-trial data. For testing condition effects, we preferred to preserve the original data structure and model variation within and across dogs. Subsequently, the pooled data allowed us to test which condition effects were (un)likely artefacts of a moving slope.

Aperiodic broadband power (≤ 30 Hz) was obtained as an output of IRASA, integrated (*trapz.m*), and log-transformed.

### Data and analyses

The final data comprised 226 recordings from 14 dogs. Of these, 120 were baseline recordings. Most recordings were half a minute long: the mode and median of the distribution were at exactly 30 s (range spanned from 18 to 48 s). Since all testing situations involved withholding an appetitive response for ~ 30 s, we deem it valid to treat test trials within and across dogs as cumulative, despite using different rewards (see Supplementary Table S1). Therefore, condition was used in below analyses as a binary variable (0 = baseline, 1 = test).

We report two main approaches to analysing frequency-band power, which we regard as complementary. Analyses based on pooled data were best suited for applying slope-correction (Gerster et al. 2022), but trial-by-trial information is lost, and within-subject variation cannot be adequately modelled. Single-trial data did not have this shortcoming but was ill-suited for slope-correction. As a first test of condition effects, we therefore focused on single-trial data, seconded by a control analysis performed on the pooled data, to account for the aperiodic slope. An additional reason to combine both approaches was to optimize how exactly the slope is accounted for. Since thresholding is common in subtraction-based detrending (Cole et al. 2019) it invites a risk for double dipping (Kriegeskorte et al. 2009) if condition effects are also tested with slope-corrected power. Our analysis protocol separates accounting for the slope from testing for condition effects. Another way to achieve this was to use division-based detrending on the pooled data (Brake et al. 2024). The latter is now reported in the Supplementary, since here it is redundant and overall in agreement with the results presented in the main text.

Single-trial data were analysed in a GLMM (Generalized Linear Mixed Model) format, using subject ID and condition as a random slope factor to account for which trials belong to the same dogs. For relative power, which can be expressed as a fraction of 1, Beta regression is recommended (Geissinger et al. 2022) and was additionally supported by better model fit, i.e. lower AIC values (compared to a standard Gaussian assumption). Analyses of aperiodic broadband power used a standard Gaussian distribution assumption. All models also included trial duration as a covariate to control for variation in the length of the recordings. Alternatively, this was controlled for by strictly including only trials with 30 s length (see Supplementary control analyses for the main findings).

In a follow-up analysis, detrending the pooled data by subtraction plus thresholding (Cole et al. 2019) allowed us to test if the spectrum includes peaks that clearly stand out from the slope. According to Brake et al. (2024) the absence of clear peaks leaves open a possibility for pseudo-rhythmic activity from aperiodic sources. The threshold tests were performed using Wilcoxon signed-rank tests for one-sample (testing for each condition which frequency band power is significantly above 0).

Analyses were performed in R (Development Core Team 2021) or JASP - ’Jeffreys’s Amazing Statistical Program’ (open source), Love et al. (2019). Beta regression was implemented using the glmmTMB package (Brooks et al. 2017; McGillycuddy et al. 2025). The R formula for glmm analyses followed this structure:

  

$$model{\text{ }}< - {\text{ }}glmmTMB(d\,\sim \,C{\text{ }}+{\text{ }}\left( {C|id} \right)\,+\,t,{\text{ }}family\,=\,x)$$

Where *d* is the dependent variable, *C* is the condition, *id* is the subject identification string, *t* is trial duration and *x* is the chosen distribution assumption. While we report primarily the nominal significance in the result section, we also address the implications of adjusting for multiple comparisons in the discussion, given that a variation of this model was run ten times (2 × 5, twice for each electrode and for 5 dependent variables: theta, alpha, beta, delta, broadband power).

Figures were plotted with Matlab and GraphPad Prism.

## Results

### Behaviour

The dogs mostly succeeded in following the instructions of the test condition, and waited for ~ 30 s until permission was given. This was sufficient for obtaining a minimum of 6 valid test trials with each animal. Twice, a reward was prematurely consumed within the first few seconds (both times by the same dog, ASS). These trials were discarded before analysis.

### Frequency-power

#### Condition effects (single trial data)

On Fz there was a significant condition effect (test > baseline) for theta power (glmm: β = 0.206, 95% CI: 0.064–0.347, z = 2.852, *P* = 0.004), alpha power (glmm: β = 0.236, 95% CI: 0.079–0.394, z = 2.935, *P* = 0.003), and beta power (glmm: β = 0.289, 95% CI: 0.019–0.560, z = 2.096, *P* = 0.036). An inverse condition effect (test < baseline) was observed for delta power (glmm: β = -0.272, 95% CI: -0.457 - -0.087, z = -2.879, *P* = 0.004). No condition effects were observed on Cz for alpha, delta, or theta (glmm: *P* > 0.1), but there was an effect (test > baseline) for beta (glmm: β = 0.250, 95% CI: 0.006–0.494, z = 2.010, *P* = 0.044).

#### Visual inspection and threshold testing (pooled data)

On Fz, spectral power was significantly above the 1/f slope for theta in the testing condition (W = 36, *P* = 0.014) and delta in both conditions (baseline: W = 105, *P* < 0.001; test: W = 105, *P* = 0.001). For other band-condition combinations on Fz, power was below the threshold (*P* > 0.1). On Cz, spectral power was above the threshold in both conditions, for theta (both conditions: W = 21, *P* = 0.036) and delta (baseline: W = 105, *P* < 0.001; test: W = 91, *P* = 0.002), but not for other bands (*P* > 0.1).

**Fig. 3:**
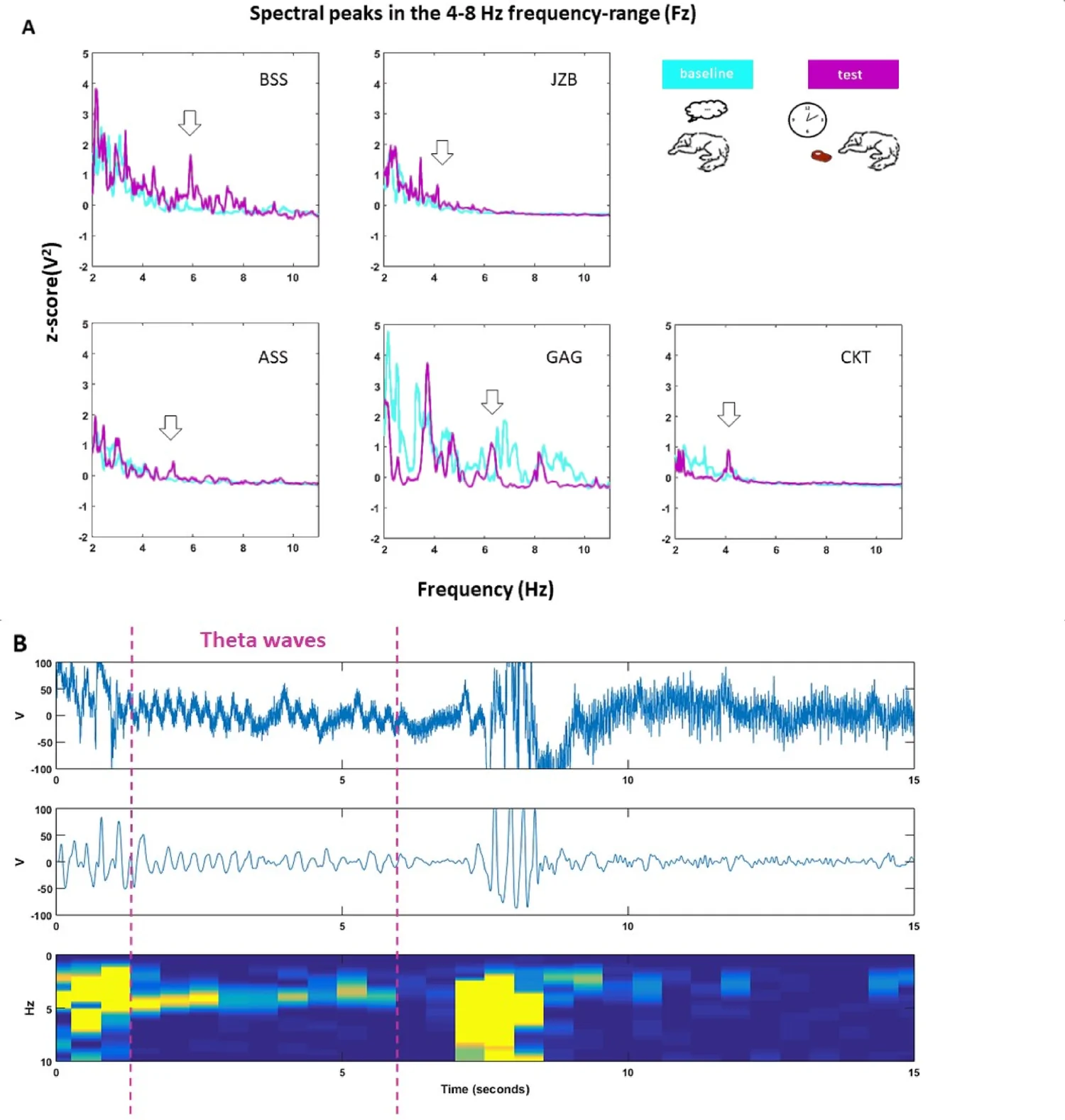
Visible, localized peaks in the 4–8 Hz frequency range for median-averaged and standard score transformed spectra in the case of 5 dogs (**A**). For each subplot, x-axis: frequency (Hz), y-axis: standard-score transformed power from the control condition (light blue) and test condition (purple). Peak visibility overlapped with non-zero power above the 1/f adjusted threshold. Example illustration of theta-waves in the time-domain (**B**). Example image is taken from a single test trial of JZB (first 15 s), top to bottom: raw signal (only corrected for eye-movements), filtered trace (passband: 3–10 Hz), time-frequency plot with heat map (displayed color range corresponds to 0–100 W/Hz). The period of increased theta activity is emphasized by framing it between two vertical dashed lines

In 5 of 8 dogs, for which power in the 4–8 Hz was above the threshold (> 0), we also observed distinguished, localized peaks in the theta frequency range. These are shown in Fig. 3.

### Aperiodic broadband power

A significant condition effect on aperiodic broadband power (test < baseline) was observed on Fz (glmm: β = -0.656, 95% CI: -1.026 - -0.286, z = -3.474, *P* < 0.001) and Cz (β = -0.601, 95% CI: -1.134 - -0.067, z = -2.205, *P* = 0.028).

### Relationship between broadband power reduction and theta elevation

**Fig. 4:**
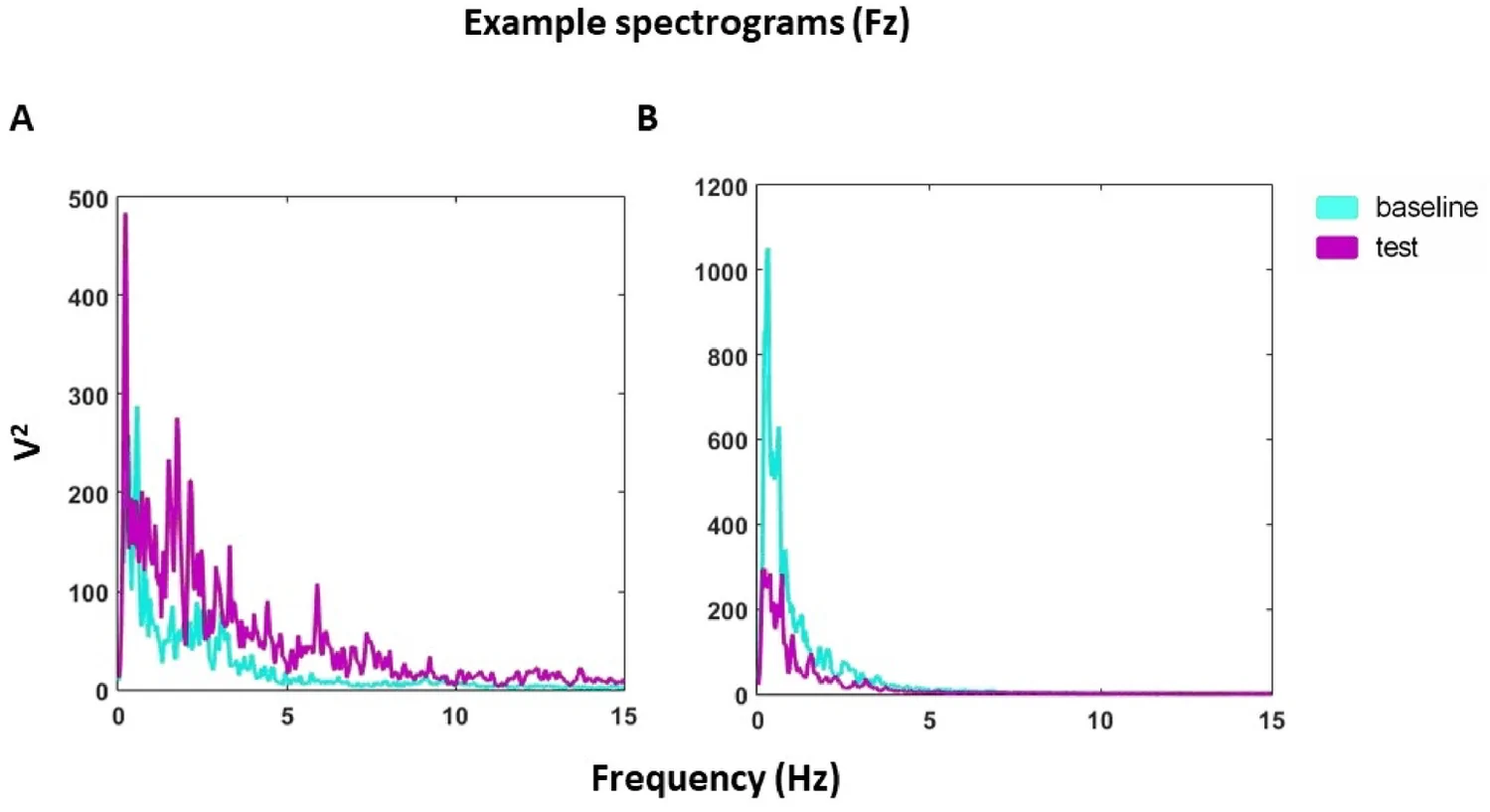
Median-averaged (across trials) example spectra for two dogs, exemplifying a distinct theta frequency peak in the test condition for BSS (**A**) and a flattened 1/f slope with reduced broadband power in BAA (**B**). For each subplot, x-axis: frequency (Hz), y-axis: power from the control condition (light blue) and test condition (purple). Although both changes were found significant on the group level, they were not observed together in the median spectra, and were mostly dissociated on the level of single-subject data (see single-subject tests)

Although both aperiodic broadband power and theta-band elevation reached significant condition effects on the group level, they were not visible in the same median-averaged spectra (see examples in Fig. 4). We tested this statistically by correlating the magnitude of broadband power reduction (baseline - test) to the magnitude in visible theta elevation (test - baseline) in the pooled data. Broadband power reduction and theta elevation were negatively correlated on Fz (*r* = -0.584, *P* = 0.024), but not on Cz (*P* = 0.455).

## Discussion

This work represents a first inquiry into the utility of EEG for studying cognitive control in dogs. In subjects who had to withhold approaching a within-reach reward for ~ 30 s, we observed two concomitants of cognitive control - elevated frontal midline theta and reduced aperiodic broadband power, both of which mirror human findings (Hummel et al. 2002; Inanaga 1998; Lu 2025; Michel and Brunet 2019; Ullsperger et al. 2014; van Schie et al. 2022; Zavala et al. 2018). While there is some debates in the literature on the utility of adjusting for multiple comparisons (Gelman et al. 2012; Perneger 1998), we note that under an adjusted alpha level (*P* = 0.005) our main findings pertaining to theta and aperiodic broadband power still remain significant.

Among frequency-specific EEG signatures of control, only theta power departed significantly from the underlying aperiodic slope. Other concomitants of cognitive control reported in the literature, like alpha and beta power (Hummel et al. 2002; Hwang et al. 2014; Lu et al. 2024; Zavala et al. 2018) failed the threshold test. Beta power would also not have survived an adjustment for multiple comparisons (adjusted alpha level at *P* = 0.005). We therefore below primarily discuss the implications of theta and broadband condition effects.

A task-related decrease in aperiodic broadband power suggests a flattening of the 1/f slope. Aperiodic slope, intercept and broadband power all describe aspects of aperiodic activity which have emerged as relatively new, but promising correlates of mental effort (Jacob et al. 2021; Kardan et al. 2020; Lu et al. 2024; Pei et al. 2023). The reduction in broadband power by itself, without consideration for the slope and its intercept, has proven most informative in humans (Lu 2025) with regard to decoding mental effort. It was reduced by the delayed gratification task in our dogs, as well. Since this was a within-subject effect we can exclude age (Voytek et al. 2015) as an alternative explanation. Another possible concern is that 1/f activity changes can reflect general shifts in arousal and wakefulness (Lendner et al. 2020; Medel et al. 2023). However, there is preliminary evidence that the changes in aperiodic activity observed here could be somewhat localized - the effect was stronger over Fz than Cz. The reason this matters is that aperiodic broadband power is inversely related to BOLD activation in fMRI (Jacob et al. 2021) and that makes the more frontally pronounced decrease in broadband power observed here align with the increased frontal lobe BOLD response, which Cook et al. (2016b) reported for dogs engaged in a version of the Go/No-Go task. The most likely physiological interpretation of the aperiodic component is, however, not conclusively agreed upon (Donoghue et al. 2020) and can be expected to vary between contexts (Brake et al. 2024).

There is stronger evidence for a localization of the theta effect. Task-related theta elevation in the current study was pronounced over Fz. The relevance of topography in dog EEG data is supported by previous work, showing that humanlike differences between frontal and central recording sites can be replicated in dogs (Iotchev et al. 2019), although only two midline electrodes are commonly used in this species (Kulgod et al. 2025), as was also done here.

In five dogs (BSS, JZB, ASS, GAG, CKT), we observed distinguishable, localized peaks in the 4–8 Hz theta range. The peaks were either exclusive to the test condition or (for one dog) present in both. As an objective support for this observation, theta power was above threshold (1 standard deviation above the slope) in all dogs for which theta elevation was visible in the median-averaged spectrum. Alpha band power (which overlaps with theta around 8 Hz) was not significantly above threshold (on the group-level). In rare cases for which alpha exceeded the 1/f threshold within a dog (see shared data), it formed a separate, from theta, peak (see e.g. GAG in Fig. 3). This suggests humanlike band-limits for theta and adds to a growing body of evidence, suggesting that most EEG patterns share a similar frequency signature in humans and dogs, even when they are known (or suspected) to vary between humans and other species. Spike-wave discharges, for example, the EEG expressions of absence seizures, are well-known to display a higher frequency in WAG/Rij rats, although they provide an otherwise strong model for human spike-wave complexes (Coenen and van Luijtelaar 2003). Sleep spindles, on the other hand, for several animal species are suspected to be of much lower frequency than in humans, based on observational data (Nicol et al. 2000; Voirin et al. 2014). In dogs, both spike-wave discharges and sleep spindles are of the same frequency as in humans (Iotchev and Kubinyi 2021; Musteata et al. 2023; Paterson et al. 2026; Poma et al. 2010; Wielaender et al. 2017) and so are the frequency profiles of the different vigilance states, i.e. wakefulness, REM and deep sleep (Kis et al. 2014). The observations made here add theta oscillations to this list.

Notably, our two main effects were not seen together on the median-averaged spectra (example in Fig. 4), although both were significant on the group level. They were also negatively correlated on Fz, that is - smaller decreases in aperiodic broadband power were linked to larger theta values above threshold. This carries interesting implications. The amplitude and slope of the aperiodic signal are known to reflect changes in states of vigilance and consciousness (Medel et al. 2023), while theta rises both with sleep pressure and mental effort. These effects add up to produce even stronger theta waves when difficult tasks are performed under sleep pressure (Snipes et al. 2022). It is thus possible, and important for future work, that theta might reflect effort in the face of difficulty to maintain alertness.

A few important caveats and limitations also need to be discussed, as well as how our analysis and arguments account for these problems. While for some research questions, stimulus consistency is crucial, in our case, it was important to depart from this standard. Stimulus-consistent tests do not help differentiate stimulus-driven from condition-driven changes in EEG power. In our case the condition was not defined by stimulus identity, but by subjective stimulus value. If the stimulus is close to emotionally neutral, lower theta-power elevations during inhibitory control challenges could be expected (Andreu et al. 2019), which, in turn, would decrease the signal-to-noise ratio for one of our main variables of interest. Our choice to include toys among the possible rewards accounts for the fact that individual dogs may strongly vary in their motivation for food (Bradshaw 2006; Cook et al. 2016a) and subsequently we can expect their preference for food over toys or toys over food to vary, as well. For this reason, owners were encouraged to choose the treat based on their knowledge of the dog’s preferences. The goal was to have a test stimulus that significantly increased appetitive motivation relative to baseline, such that withholding consumption would be cognitively effortful. All dogs, whose toy-trials are included in the analyses, were confirmed by the owner to have a considerable preference towards the toy used in the test. Importantly, excluding subjects who used toy trials did not alter the results for frontal midline theta and aperiodic broadband power (see Supplementary).

One concern with using passive wakefulness as a control condition is to capture condition differences that rather reflect a drop in vigilance during trials where nothing is happening. In this regard, it is important that our baseline recordings come both from before the dog can become ‘aware’ of a task (prior to a first test recording) and also from in-between test trials. The proportion of baselines recorded before versus in-between test trials did not seem to affect individual results (compare Table 2 and single subject tests). We could not record usable EEG exactly during reward consumption since chewing food or grabbing a toy comes with strong movement artefacts, it may therefore be advisable to explore paradigms in the future that avoid consumable rewards altogether in order to create even stronger controls and rather compare simple versus difficult rules in the execution of trained tasks.

Dogs’ waking EEG is inherently more noisy than that of humans (Eleőd et al. 2023), and this has led to a relatively large portion of EEG studies in dogs to focus on sleep physiology (Kulgod et al. 2025), when the animals are relatively motionless. This is not to say that awake EEG in the dog is falling too far behind (Bálint et al. 2022; Boros et al. 2024; Castillo et al. 2022; Folkard et al. 2024; James et al. 2017; Wrzosek et al. 2024), but the awake canine subject remains overall more challenging. We have undertaken three measures to reduce this problem. First, we adopted a simple, but well-established protocol for eye-movement correction - regression-based cleaning (Ronca et al. 2024), which allowed us to keep most of the signals, unlike the more common epoch rejection we so far applied in our sleep studies. Second, the use of medians instead of averages for pooling spectral power across trials, time windows, and frequency bins allowed us to obtain estimates that are less likely to be skewed by unique and unusual events, i.e. by artefacts which survived the initial cleaning. Finally, focusing on a question for which the topography of the response matters also makes it less likely to observe a false positive confirmation of our hypothesis, since in dogs, artefacts should affect both midline electrodes equally. To make valid comparisons between Fz and Cz frequency power changes, relative power needed to be used, since absolute power would almost certainly always be lower on Cz in all bands, as Cz is closer to the reference electrode.

As one last note of caution, the present work does not explore how individual characteristics like age, sex, neuter status, and breed may moderate the experimentally induced elevation in theta power. In dogs, age, sex, neutering and breed differences can be linked to altered EEG patterns at least during sleep (Iotchev et al. 2019, 2023). Most importantly, we know from human subjects that theta-elevation in response to cognitive load is reduced in older individuals (Darna et al. 2025) and possibly higher in young women compared to young men when elicited by oddball perceptual paradigms (Yener et al. 2024). To identify to what extent individual differences in theta power elevation reflect individual differences in the capacity for self-control, these variables must be accounted for in the future. This is, however, a follow-up question outside the scope of this study.

How do the presented findings connect with previous work and possible future efforts specifically regarding dogs’ capacity for cognitive control? Cook et al. (2016b) had already demonstrated frontal lobe activation, with the fMRI source localization technique. However, meaningful follow-up work for their exciting result is inherently limited by the cost of implementing fMRI (Montgomery 2023), and the limited space within the scanner allows for little more than passive perception experiments, which dominate the literature on dog fMRI (Andics et al. 2016; Berns et al. 2012; Gábor et al. 2020; Thompkins et al. 2021). The use of a more natural setting allows us to derive more realistic assumptions, as to when the animals make use of their ‘higher’ faculties. It is clear from previous work that dogs’ use of inhibitory self-control is in many respects not on par with that of humans. For example, dogs tend to continue to approach a wrong target in two-choice tasks, even when a reduced latency towards the right choice betrays that they are learning the difference (Bolló et al. 2025; Piotti et al. 2018). On the other hand, our results suggest that common situations for dogs, like abstaining from approaching or grabbing what the owner has forbidden, are possibly solved by the use of cognitive control. This is contrary to the claim that most of dogs’ apparent cognitive behaviours can be reduced to instances of conditioning alone (Wynne 2016).

On a more practical note, identifying neuro-cognitive mechanisms of self-control around food can have important implications for animal welfare, as obesity among family dogs is an increasing problem (Lund et al. 2006). Poor cognitive control is an important comorbidity of obesity in humans (Guxens et al. 2009; Kamijo et al. 2014; Sellaro and Colzato 2017), while also linked to other complications, e.g. ADHD (de Zeeuw et al. 2012), and depression (Grahek et al. 2019). These are conditions also of interest to the veterinary practitioner (Kealy et al. 2002; Laflamme 1997; Senay 1966). Therefore, more intensive research into dogs’ cognitive control capacities could have practical implications for the veterinary field, while also strengthening the dog as an emerging model for human ADHD (Bunford et al. 2019; Kovács et al. 2025; Vas et al. 2007) and depression (Senay 1966).

Our findings help set the stage for studying canine cognitive control across different behavioural paradigms and neuro-imaging modalities, therein complementing the first steps taken by Cook et al. (2016b) in the direction of establishing a canine cognitive neuroscience. As a note of caution, theta power does not distinguish well between types of mental effort (Cavanagh and Frank 2014; Eisma et al. 2021; Inanaga 1998; Michel and Brunet 2019; Ullsperger et al. 2014). It also does not support a finer distinction among the more specialized structures within the frontal lobes (Cavanagh and Frank 2014; Manza et al. 2016; Roelofs et al. 2006; Swick et al. 2008). A more frontally pronounced theta-elevation, as observed here, can however be distinguished from the more diffuse activation seen in drowsiness (Britton et al. 2016; Snipes et al. 2022) and is more likely from a source broadly within the frontal lobes. In fact, some seemingly separate forms of cognitive control have been localized to the same substructure within the frontal network, e.g. the anterior cingulate cortex has been argued to support inhibitory control (Albert et al. 2011; Manza et al. 2016), conflict monitoring (Carter and Botvinick 1999), and anticipatory control (Roelofs et al. 2006). Crucially, a humanlike, frontally pronounced theta-elevation as observed here, does not align with alternative scenarios that would fully exclude cognitive effort: simply registering the presence of reward is linked to changes in higher frequency bands, such as beta and gamma (Marco-Pallares et al. 2015). Pure arousal i.e. waking up from a more drowsy, idle state, should lead to less, not more theta power (Britton et al. 2016; Snipes et al. 2022). Finally, reward expectation, unlike passive reward registration, is part of anticipatory control (Chiew and Braver 2014). Overall, the data aligns with a transition from low to high cognitive and attentional engagement, as the animals switch from baseline to test. Future efforts must aim to further distinguish between facets of cognitive control and the finer anatomical structures involved. What the present results show is that an objectively demanding situation, requiring restraint and patience in the face of luring provocation, can modify the same EEG parameters in dogs that correspond in humans to more attentive and cognitive engagement with the environment.

## Supplementary Information

Below is the link to the electronic supplementary material.

Supplementary Material 1

## Data Availability

The data used in this study is shared on Open Science Framework (OSF) under this link: [https://osf.io/9efcs/overview? view_only=4c05719bb6234dd9aab91f420821f306](https:/osf.io/9efcs/overview? view_only=4c05719bb6234dd9aab91f420821f306).

## Footnotes

1 But with a different thematic focus: sleep-dependent learning.

2 The relationship between frequency bands was tested across trials using a Generalized Linear Mixed Model approach to account for which trials belong to the same dogs. Hence this was not a correlation in the classical (Pearson) sense.
