# Supplementary material for: A human-like EEG signature of cognitive control in the domestic dog
Source: Anim Cogn. 2026 Aug 11;29:81. doi: 10.1007/s10071-026-02096-8 (PMC13748066; doi:10.1007/s10071-026-02096-8)
Supplement: Supplementary file 1 [file 10071_2026_2096_MOESM1_ESM.docx]

A human-like EEG signature of cognitive control in the domestic dog

Ivaylo Borislavov Iotchev^*1^, Anna Kis^1^, Márta Gácsi^2,3,4^

^1^ „Momentum” Psychobiology Research group, HUN-REN Research Centre for Natural Sciences, Institute of Cognitive Neuroscience and Psychology, 1117 Budapest, Hungary

^2^ Department of Ethology, ELTE Eötvös Loránd University, Pázmány Péter sétány 1/c, 1117 Budapest, Hungary

^3^ HUN-REN–ELTE Comparative Ethology Research Group, Budapest, Hungary
^4^ NAP Canine Brain Research Group, ELTE Eötvös Loránd University, Budapest, Hungary

*corresponding author, email: [ivaylo.iotchev@gmail.com](mailto:ivaylo.iotchev@gmail.com)

**Supplementary**

Table S1. Type of reward and cross-trial consistency (i.e. was an identical treat used on all trials) for each dog in the experiment.

| **Dog ID** | **Type** | **Same across trials** |
| --- | --- | --- |
| BSS | ham | yes |
| FMG | sausage | yes |
| DMG | sausage | yes |
| JZB | special snack | yes |
| VLZ | special snack | yes |
| ASS | special snack, ball | no |
| KAJ | special snack (various) | no |
| GAG | stuffed animal | yes |
| FAG | special snack | yes |
| CKT | special snack | yes |
| NKT | special snack | yes |
| BCC | ham | yes |
| KJA | special snack (various) | no |
| BAA | special snack (various) | no |

**Absolute power exploration**

We used a glmm format to analyse how absolute power is ‘correlated’ between frequency bands (alpha, beta, delta, theta), specifying subject ID as a random factor to account for observations (trials) that come from the same dogs. This analysis happened at an earlier stage of our work. Therefore, it deviates from our main analyses in a few choices preceding statistical testing, as well as the format in which it is reported. Specifically, frequency resolution was set somewhat lower (0.1 instead of 0.01 Hz). Also the boundaries of the frequency bands were somewhat shifted to avoid overlap in the definitions, i.e. theta was defined as 5-7 Hz (instead of 4-8 Hz) and beta as 13-30 Hz (instead of 12-30 Hz). This changed after our visual inspection of the data (and backed by the more objective threshold testing) suggesting that canine theta might indeed be better captured by the ‘full’ 4-8 Hz range. Overall, while we do not expect these differences to meaningfully affect the results, for consistency we report this preliminary analysis here, instead of in the main text:

Absolute power was significantly and positively correlated between any two frequency bands, on Fz (glmm: alpha and beta, ꭕ^2^ = 25.31, P < 0.001; glmm: alpha and delta, ꭕ^2^ = 36.87, P < 0.001; glmm: alpha and theta, ꭕ^2^ = 46.09, P < 0.001; glmm: beta and delta, ꭕ^2^ = 16.70, P < 0.001; glmm: beta and theta, ꭕ^2^ = 18.84, P < 0.001; glmm: theta and delta, ꭕ^2^ = 50.62, P < 0.001), and on Cz (glmm: alpha and beta, ꭕ^2^ = 20.84, P < 0.001; glmm: alpha and delta, ꭕ^2^ = 27.30, P < 0.001; glmm: alpha and theta, ꭕ^2^ = 45.86, P < 0.001; glmm: beta and delta, ꭕ^2^ = 16.97, P < 0.001; glmm: beta and theta, ꭕ^2^ = 20.28, P < 0.001; glmm: theta and delta, ꭕ^2^ = 47.47, P < 0.001).

Since we thus found all bands of interest to interrelate in the same direction, we also tested if the entire spectrum shifts between conditions:

The broadband^[1]^ power spectrum (1 - 30 Hz) displayed a significant effect of condition (test < baseline) on Fz (ꭕ^2^ = 6.80, P = 0.009) and Cz (ꭕ^2^ = 5.17, P = 0.023).

**Testing condition effects using division-based detrending (pooled data)**

Below analysis is considered redundant for the main text, but combines slope-correction and condition test for the same data (but without allowing us to model within-subject variation).

Because of the condition effect on broadband power, we tested condition effects on frequency power using division-based detrending (Brake et al. 2024). A significant condition effect (test > baseline) was observed on Fz for theta (W = 13, P = 0.011), but not for alpha, beta, or delta (P > 0.8). On Cz significant condition effects (test > baseline) were observed for theta power (W = 19, P = 0.035) and beta power (W = 18, P = 0.030), but not for alpha or delta power (P > 0.2).

**Excluding subjects who used toy trials**

We revisited our main results using only subjects who were uniformly tested with food items, thus excluding ASS and GAG. For frontal midline theta we still observed a significant condition effect for theta on Fz (test > baseline, glmm: β = 0.189, 95% CI: 0.024 – 0.354, z = 2.250, P = 0.024), and for aperiodic broadband power on Fz (test < baseline, β = -0.667, 95% CI: -1.047 - -0.287, z = -3.438, P < 0.001) and Cz (test < baseline, β = -0.681, 95% CI: -1.231 - -0.132, z = -2.429, P = 0.015).

**Excluding trials with length ≠ 30 seconds**

We also revisited our main findings by conducting analyses only on trials of exactly 30 second duration. These were 108 trials from 13 dogs. These models were overall identical to the ones used in the main text, except that trial duration was not included as a covariate.

We still observed a significant condition effect for theta power on Fz (test > baseline, glmm: β = 0.224, 95% CI: 0.092 – 0.355, z = 3.331, P = 0.001) and aperiodic broadband power on Fz (test < baseline, glmm: β = -0.702, 95% CI: -1.165 - -0.239, z = -2.970, P = 0.003) and Cz (test < baseline, glmm: β = -0.707, 95% CI: -1.274 - -0.140, z = -2.444, P = 0.015).

1. This is not the aperiodic broadband used later in our formal analyses, but a total sum of power across bands (alpha, beta, delta, theta).
